# Supplementary figures and images for: Revisiting the type material of two African Diplozoinae (Diplozoidae: Monogenea), with remarks on morphology, systematics and diplozoid specificity
Source: PeerJ. 2024 Feb 28;12:e17020. doi: 10.7717/peerj.17020 (PMC10908257; doi:10.7717/peerj.17020)

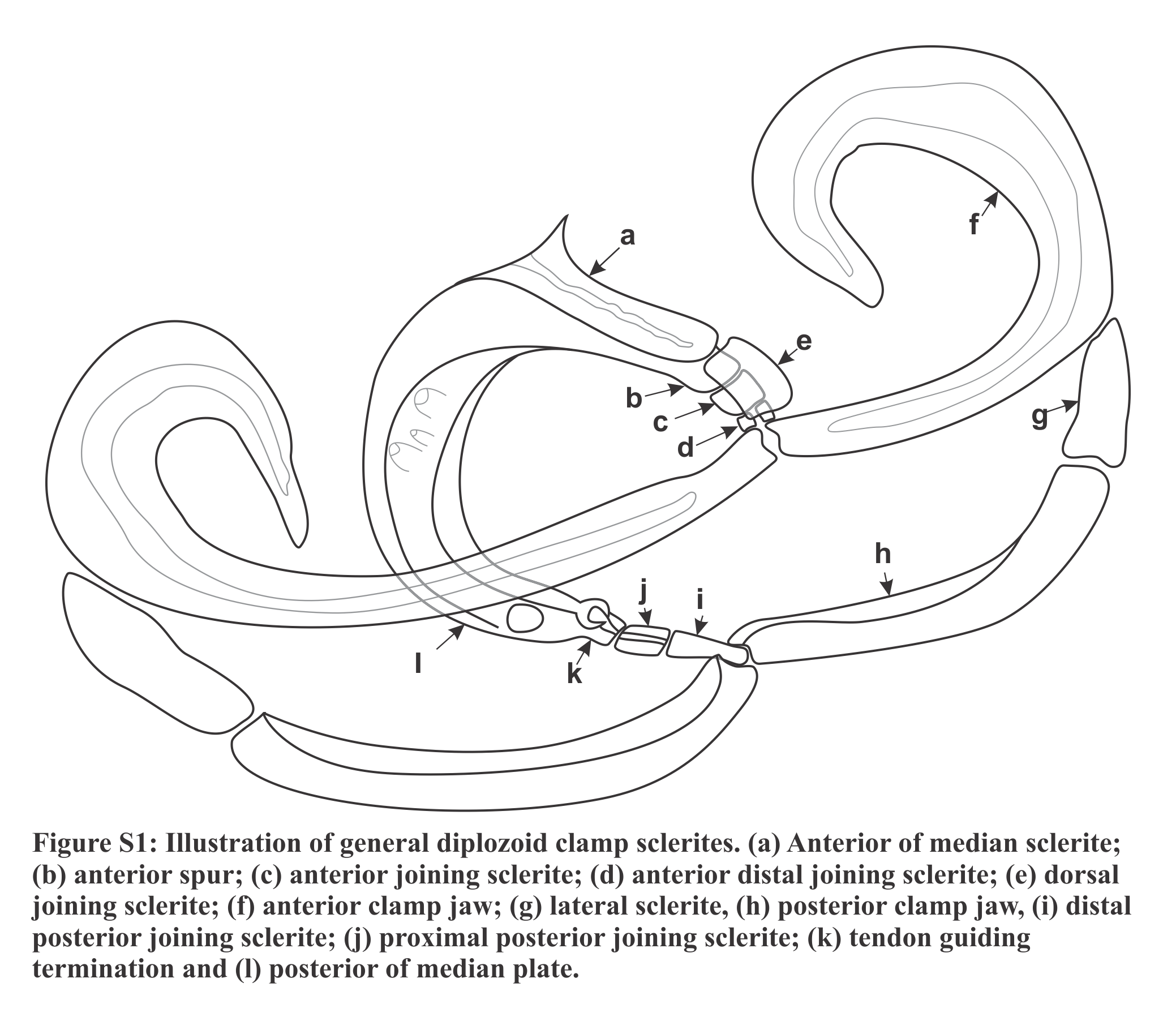

Supplement: Supplemental Information 3 [file peerj-12-17020-s003.png]

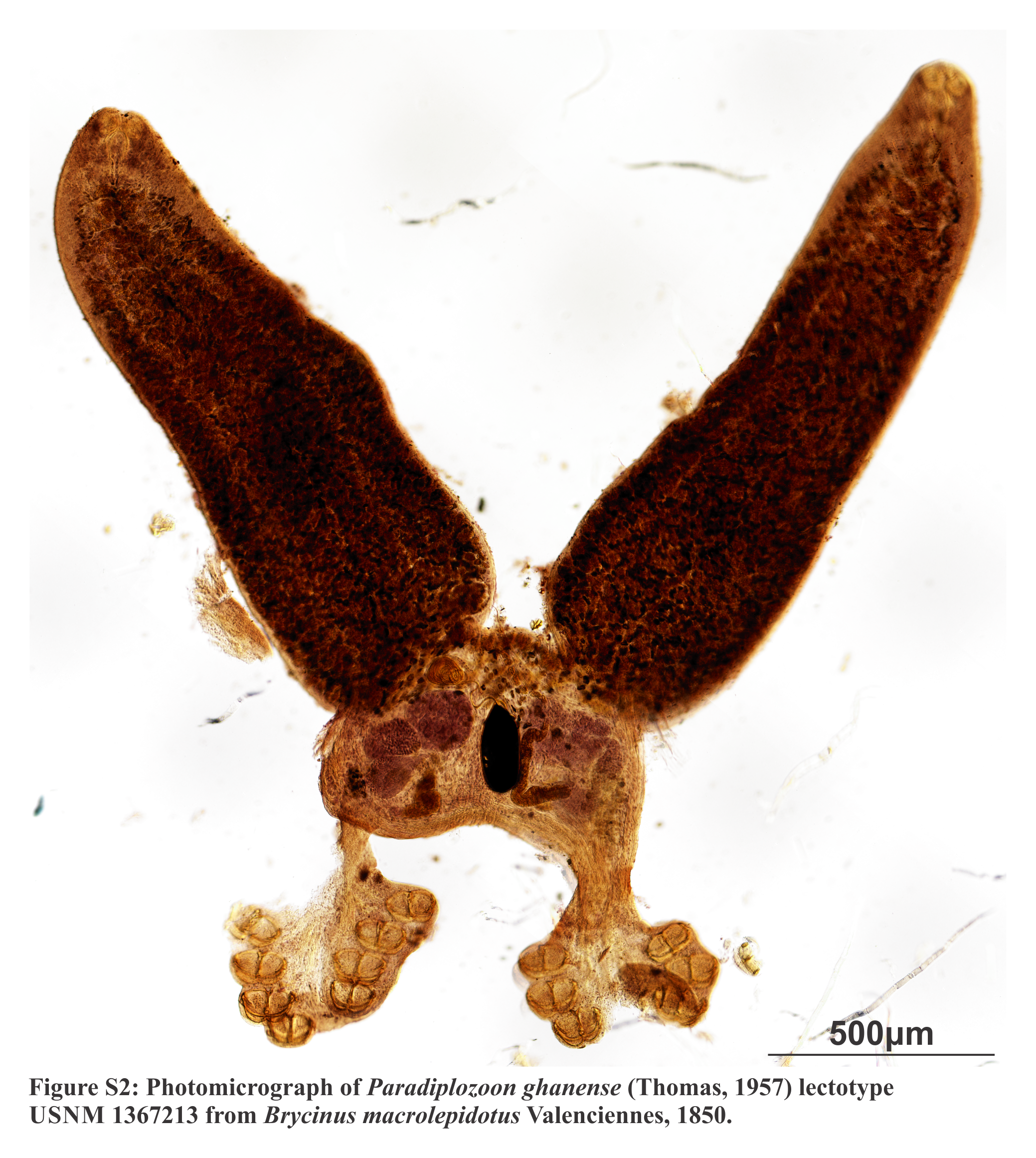

Supplement: Supplemental Information 4 [file peerj-12-17020-s004.png]

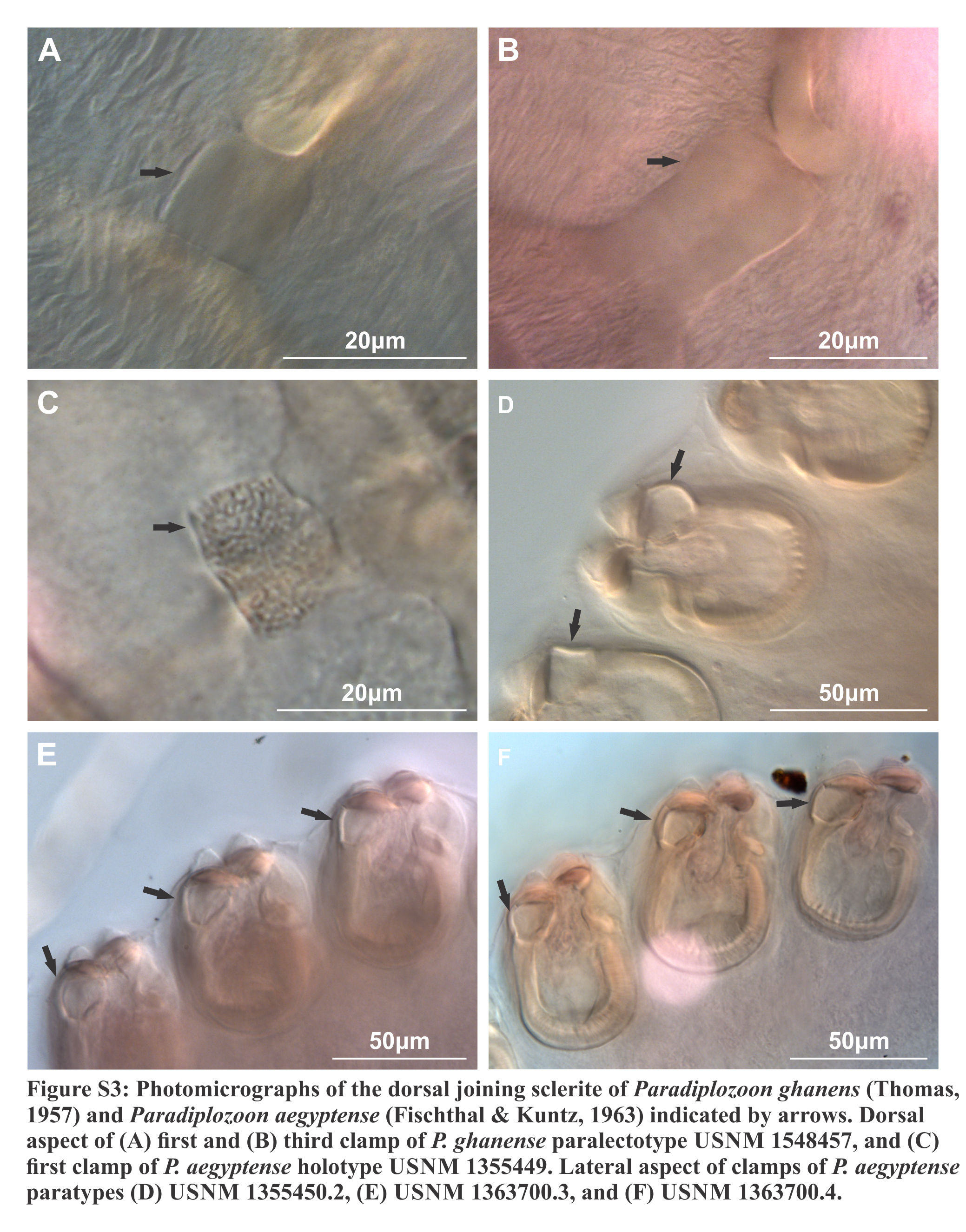

Supplement: Supplemental Information 5 [file peerj-12-17020-s005.png]

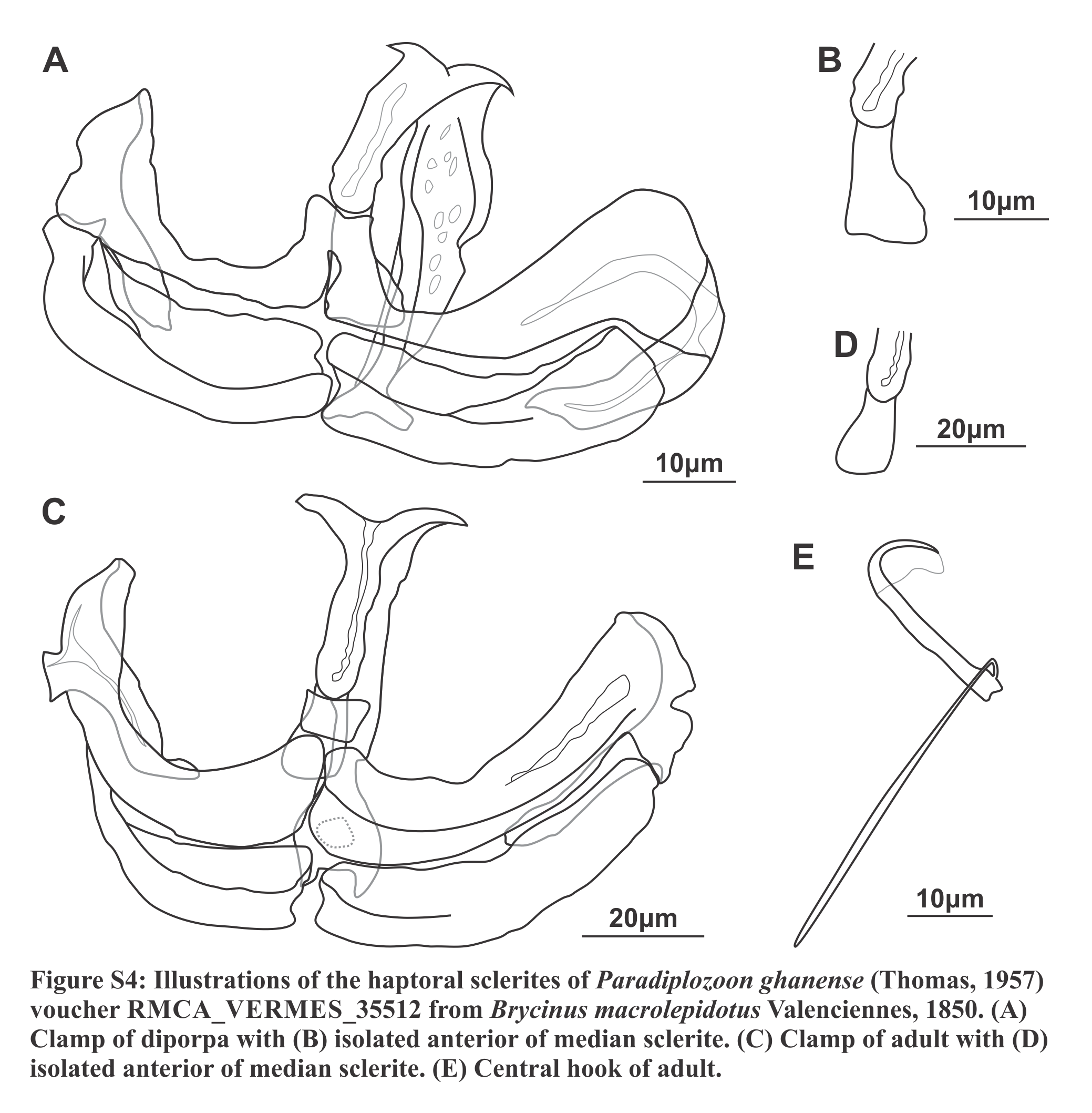

Supplement: Supplemental Information 6 [file peerj-12-17020-s006.png]

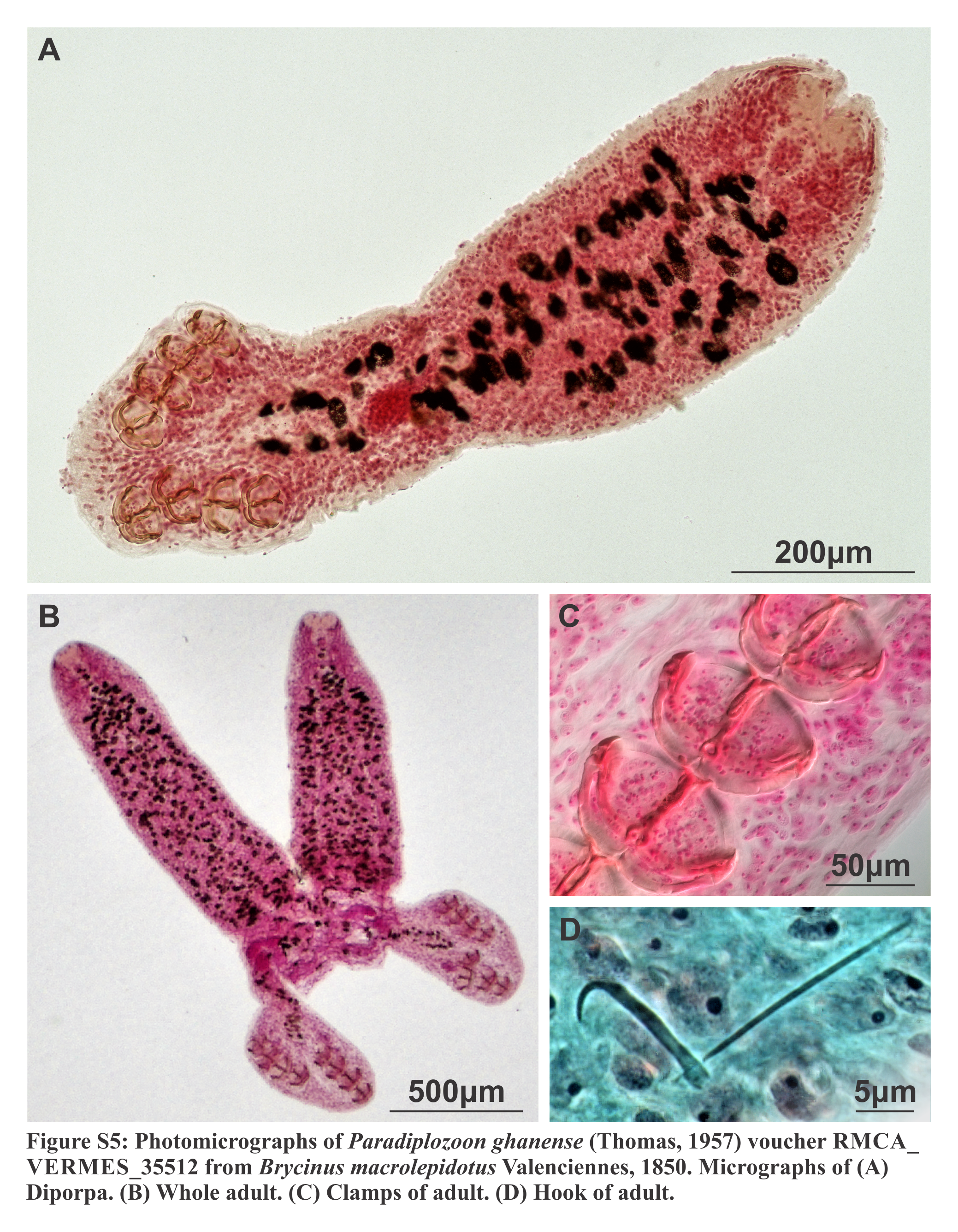

Supplement: Supplemental Information 7 [file peerj-12-17020-s007.png]

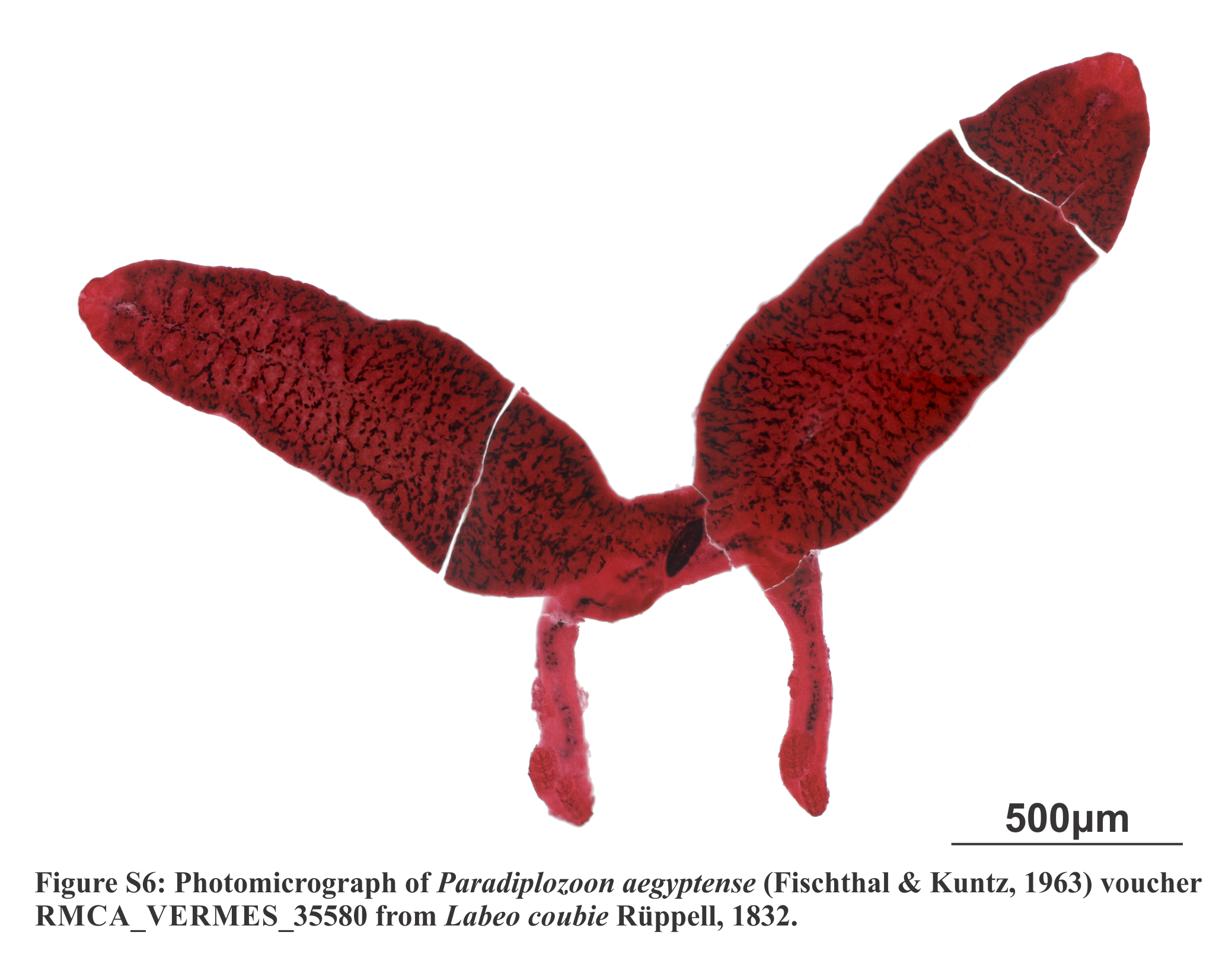

Supplement: Supplemental Information 8 [file peerj-12-17020-s008.png]
